# Aza-Triangulene: On-Surface Synthesis and Electronic and Magnetic Properties

**DOI:** 10.1021/jacs.1c12618

**Published:** 2022-03-07

**Authors:** Tao Wang, Alejandro Berdonces-Layunta, Niklas Friedrich, Manuel Vilas-Varela, Jan Patrick Calupitan, Jose Ignacio Pascual, Diego Peña, David Casanova, Martina Corso, Dimas G. de Oteyza

**Affiliations:** † Donostia International Physics Center, 20018 San Sebastián, Spain; ‡ Centro de Fisica de Materiales CFM/MPC, CSIC-UPV/EHU, 20018 San Sebastián, Spain; § CIC NanoGUNE BRTA, 20018 San Sebastián, Spain; ∥ Centro Singular de Investigación en Química Biolóxica e Materiais Moleculares (CiQUS) and Departamento de Química Orgánica, Universidade de Santiago de Compostela, 15782 Santiago de Compostela, Spain; ⊥ Ikerbasque, Basque Foundation for Science, 48009 Bilbao, Spain; # Nanomaterials and Nanotechnology Research Center (CINN), CSIC-UNIOVI-PA; 33940 El Entrego, Spain

## Abstract

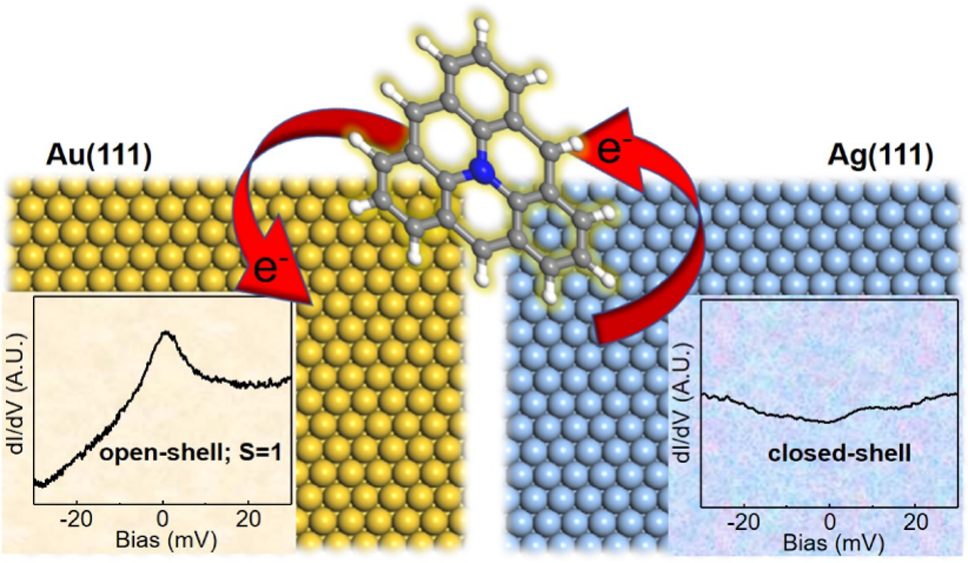

Nitrogen heteroatom doping into a triangulene molecule allows tuning its magnetic state. However, the synthesis of the nitrogen-doped triangulene (aza-triangulene) has been challenging. Herein, we report the successful synthesis of aza-triangulene on the Au(111) and Ag(111) surfaces, along with their characterizations by scanning tunneling microscopy and spectroscopy in combination with density functional theory (DFT) calculations. Aza-triangulenes were obtained by reducing ketone-substituted precursors. Exposure to atomic hydrogen followed by thermal annealing and, when necessary, manipulations with the scanning probe afforded the target product. We demonstrate that on Au(111), aza-triangulene donates an electron to the substrate and exhibits an open-shell triplet ground state. This is derived from the different Kondo resonances of the final aza-triangulene product and a series of intermediates on Au(111). Experimentally mapped molecular orbitals match with DFT-calculated counterparts for a positively charged aza-triangulene. In contrast, aza-triangulene on Ag(111) receives an extra electron from the substrate and displays a closed-shell character. Our study reveals the electronic properties of aza-triangulene on different metal surfaces and offers an approach for the fabrication of new hydrocarbon structures, including reactive open-shell molecules.

## Introduction

**Figure 1:**
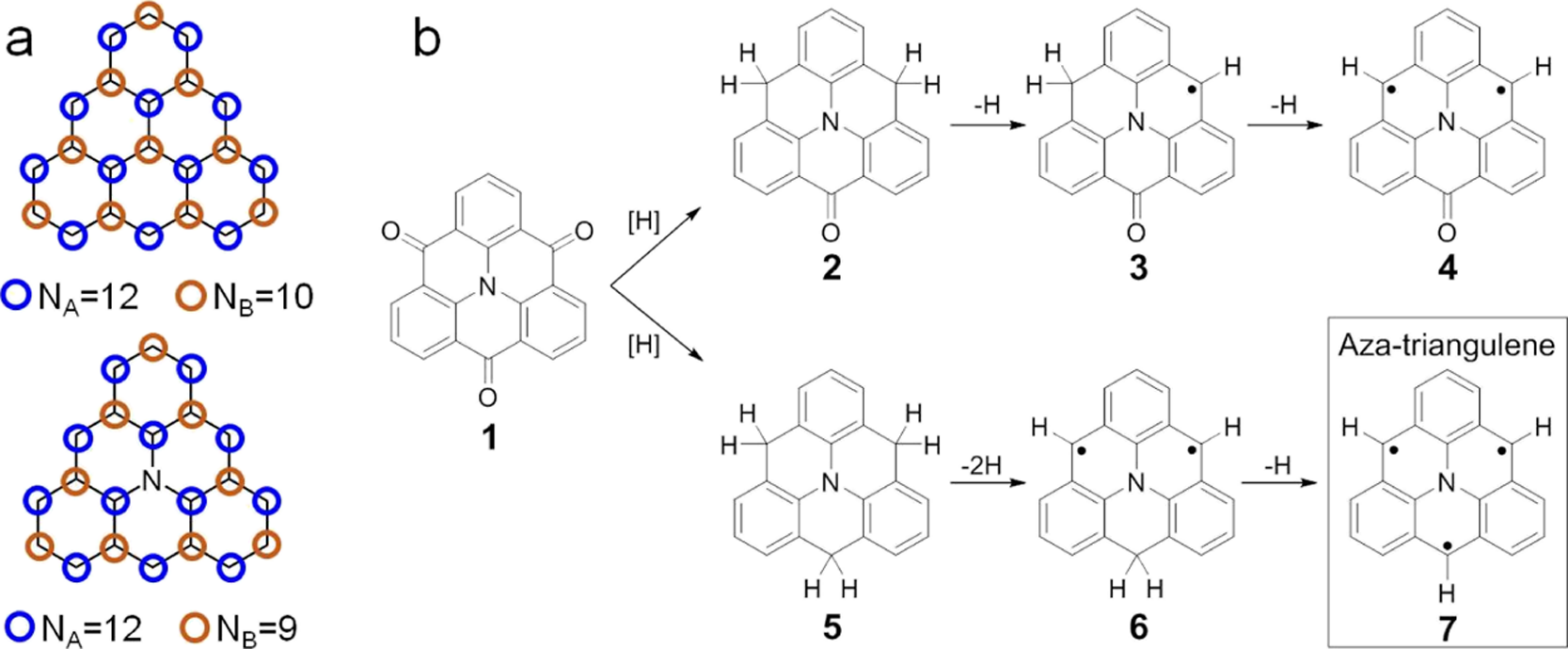
(a) Schematic representation of carbon atoms belonging to each of the two sublattices. (b) Reaction steps for the synthesis of aza-triangulene **7** on Au(111), starting from the hydrogenation of precursor **1**, followed by 250 °C annealing and subsequent tip manipulation. The final product, intermediates, and by-products observed in experiments are presented.

Triangulene, as the smallest triplet-ground-state polybenzenoid, has attracted intensive attention since it was theoretically devised back in 1953.^1^ In spite of its even number of carbon atoms, it is not possible to pair up all of its π-electrons to form a closed-shell structure.^2−4^ The total net spin of triangulene in its ground state is quantified by Ovchinnikov’s rule^5^ and Lieb’s theorem^6^ for bipartite lattices: *S* = (*N*_A_–*N*_B_)/2, where *N*_A_ and *N*_B_ denote the numbers of carbon atoms belonging to each of the two sublattices (*N*_A_ = 12, *N*_B_ = 10, and *S* = 1, Figure 1a). Due to its high reactivity stemming from its unpaired electrons, the synthesis of triangulene by conventional solution-phase chemistry has been inaccessible.^1,4^ Fortunately, the recently developed on-surface synthesis (OSS)^7−10^ under ultra-high vacuum conditions opens a door for the fabrication of reactive carbon-based structures holding π-radicals, where a rationally designed precursor is annealed at high temperatures over a catalytic surface in order to form the target product.^11−13^ Using the OSS strategy, triangulene and other extended [*n*]triangulenes (*n* > 3) have been successfully synthesized, whose structures were characterized precisely with the aid of bond-resolving scanning tunneling microscopy (BR-STM) and noncontact atomic force microscopy techniques.^3,14−17^ In addition, the open-shell character of triangulene and its derivates was confirmed by the observation of singly occupied/unoccupied molecular orbitals (SOMOs/SUMOs),^14,15^ Kondo resonances,^18^ and spin-flip excitations.^17−19^

Heteroatom doping can substantially modify the electronic and magnetic properties of graphene-based structures.^20−26^ For example, the substitution of the central carbon atom of triangulene by a nitrogen adds a π-electron to the system, resulting in different ground-state spin multiplicities with respect to that of undoped triangulene. In a naïve picture, one may assume a double occupancy of the p_*z*_ orbital on the central nitrogen atom of aza-triangulene. This would remove its contribution to the *N*_B_ count in Ovchinnikov’s rule and result in a quartet ground state (*N*_A_ = 12, *N*_B_ = 9, and *S* = 3/2, Figure 1a). The same may be expected from the chemical structure drawing, as shown in structure **7** (Figure 1b), representing a molecule with *D*_3*h*_ symmetry and three radicals, one on each molecular side. All are located on the same sublattice and are thus expected to align ferromagnetically. However, theoretical calculations predict that for aza-triangulene,^27^ a doublet ground state (*S* = 1/2) with a *C*_2*v*_ molecular symmetry, driven by a Jahn–Teller distortion,^28^ is energetically more favorable than the more intuitive *D*_3*h*_ structure.

Despite these interesting theoretical predictions,^27^ synthesis of aza-triangulene had, until now, remained elusive. Herein, we report the OSS of aza-triangulene on both Au(111) and Ag(111). Figure 1b shows the reaction procedure starting from the corresponding precursor, that is, the ketone-substituted aza-triangulene **1**. In our previous work, the combination of atomic hydrogen reduction followed by annealing was shown efficient in removing the oxygen atoms on ketone-substituted graphene nanoribbons on Au(111).^29^ Inspired by this, we employed a similar procedure for molecule **1**. Hydrogen reduction followed by annealing resulted in partial (**2**) or complete (**5**) deoxygenation of the precursor **1**. Subsequent tip-induced removal of hydrogen atoms results in byproducts **3** and **4**, intermediate **6**, and the final product aza-triangulene **7** (Figure 1b). We show that aza-triangulene donates an electron to the Au(111) substrate and bears a triplet ground state. On the contrary, aza-triangulene receives an electron from the low-work function Ag(111) surface, resulting in a closed-shell ground state.

## Results

### Synthesis

**Figure 2:**
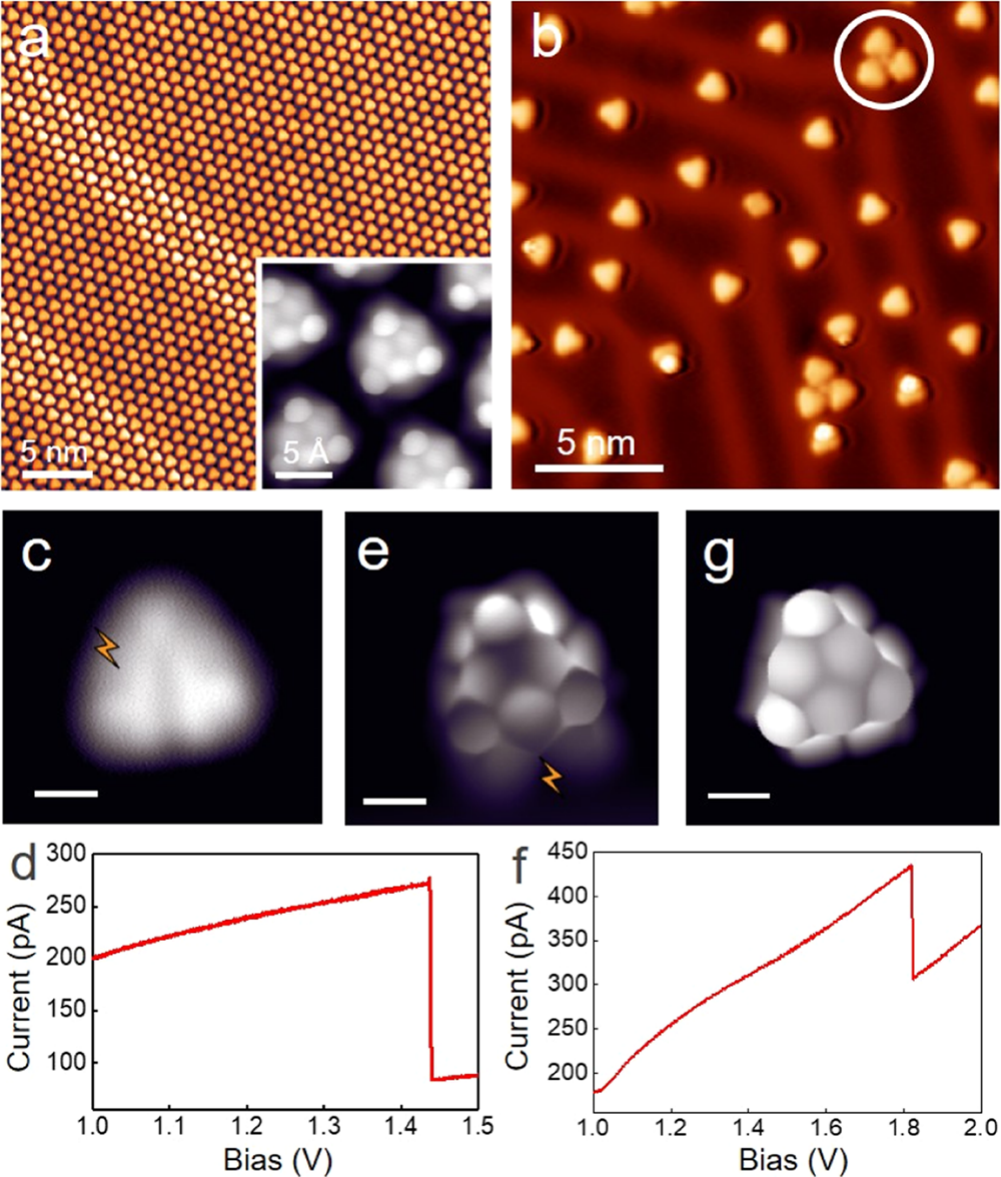
(a) STM image of the sample prepared by depositing molecule **1** on Au(111) at RT. The inset shows a BR-STM image taken by a CO-functionalized probe. (b) STM image of the sample after hydrogenation and annealing at 250 °C. A self-assembled triangulene trimer is marked with a white circle. (c,e,g) Constant-height STM images of molecules **5**, **6**, and **7**, respectively. (d,f) *I*–*V* spectroscopy demonstrating the tip-induced removal of additional hydrogens. The positions for the tip-induced pulses are marked in (c,e). Tunneling parameters: (a,b) *U* = −1 V and *I* = −100 pA; the inset of (a) and (c,e,g): *U* = 5 mV. Scale bars in (c,e,g) are 5 Å.

Precursor molecule **1** was obtained by solution-phase synthesis following a previously reported procedure (Figure S1; Supporting Information).^30−32^Figure 2a shows the STM image of the sample upon depositing **1** on Au(111) held at room temperature (RT). The molecules self-assemble into well-ordered dense islands stabilized by a two-dimensional network of intermolecular hydrogen bonds. BR-STM imaging (Figure 2a, inset) shows the ketone groups exhibiting V-shape protrusions pointing toward the hydrogen atoms of the neighboring molecules.^33^ The energy and spatial distribution of frontier molecular orbitals as obtained with scanning tunneling spectroscopy show an excellent agreement with previous reports (Figure S2).^30^

The sample held at RT was then hydrogenated by exposing to atomic hydrogen. As a result, a considerable number of sp^3^-hybridized carbon atoms were generated (Figure S3), which turned most of the molecules three-dimensional.^29^ Subsequent annealing at 250 °C caused a notable desorption and planarized most of the remaining molecular species (>70%; Figure 2b). Figure 2c shows a representative constant-height STM image of the most abundant product in Figure 2b. The removal of a hydrogen from a CH_2_ edge atom requires an annealing temperature around 300 °C,^34,35^ higher than that used to dehydrogenate the “interior” carbon atoms. Our thermal treatment therefore still maintains sp^3^-hybridized carbon atoms at the molecular edges that weaken molecule–substrate interactions,^36^ thus facilitating molecular diffusion and hindering the acquisition of BR-STM images. However, since hydrogen atoms tend to passivate the most reactive sites of nanographenes^12,37^ (which in this case are the center carbon atoms at the three zigzag edges of aza-triangulene), structure **5** is the most probable major product.

Tip-induced dehydrogenation was then employed to remove H atoms from the sp^3^-hybridized carbons of **5** (Figure 2c–f). We ramped up the bias with the tip positioned over the zigzag edge of this product (marked as a lightning in Figure 2c). The dehydrogenation is observed as a current step in *I*–*V* spectra, as shown in Figure 2d. Subsequent BR-STM images allow for the assignment of the intermediate product structure as **6** (Figure 2e). The six-membered ring containing the remaining sp^3^ carbon atom is much larger than others and exhibits a sharp corner—a widely reported fingerprint of sp^3^-carbon containing rings.^3,12,36,37^ In all our attempts, the first dehydrogenation step occurred directly from **5** to **6**, that is, by simultaneously removing two H atoms from two edges. The final product **7** was generated by the tip-induced removal of the last residual hydrogen from **6**, as confirmed by the BR-STM image in Figure 2g. It is worth mentioning that, apart from the tip manipulation approach, product **7** can also be produced directly by annealing the sample shown in Figure 2b at a temperature above 300 °C. However, because the spin density of open-shell molecules automatically boosts their reactivity,^38^ most of the molecules react and appear as dimers or oligomers, evidencing the limitations of this approach to scale up the synthesis of **7**. An example is presented in Figure S4, in which **7** and various products from molecular fusions coexist.

As marked by the white circle in Figure 2b, a few trimers with a dot in the center are also observed on this sample. These molecules retain a residual ketone group (**2**; Figure 1), and the trimer structure is apparently stabilized *via* O···Au coordination interactions and hydrogen bonds (see details in Figures S5–S7), as reported previously for other ketone-functionalized carbon nanostructures.^29,39^ Using similar tip manipulation procedures as described above, products **3** and **4** (Figure 1b) can be obtained hierarchically as well (Figures S5 and S6).

### Kondo Resonance and Charge Transfer

**Figure 3:**
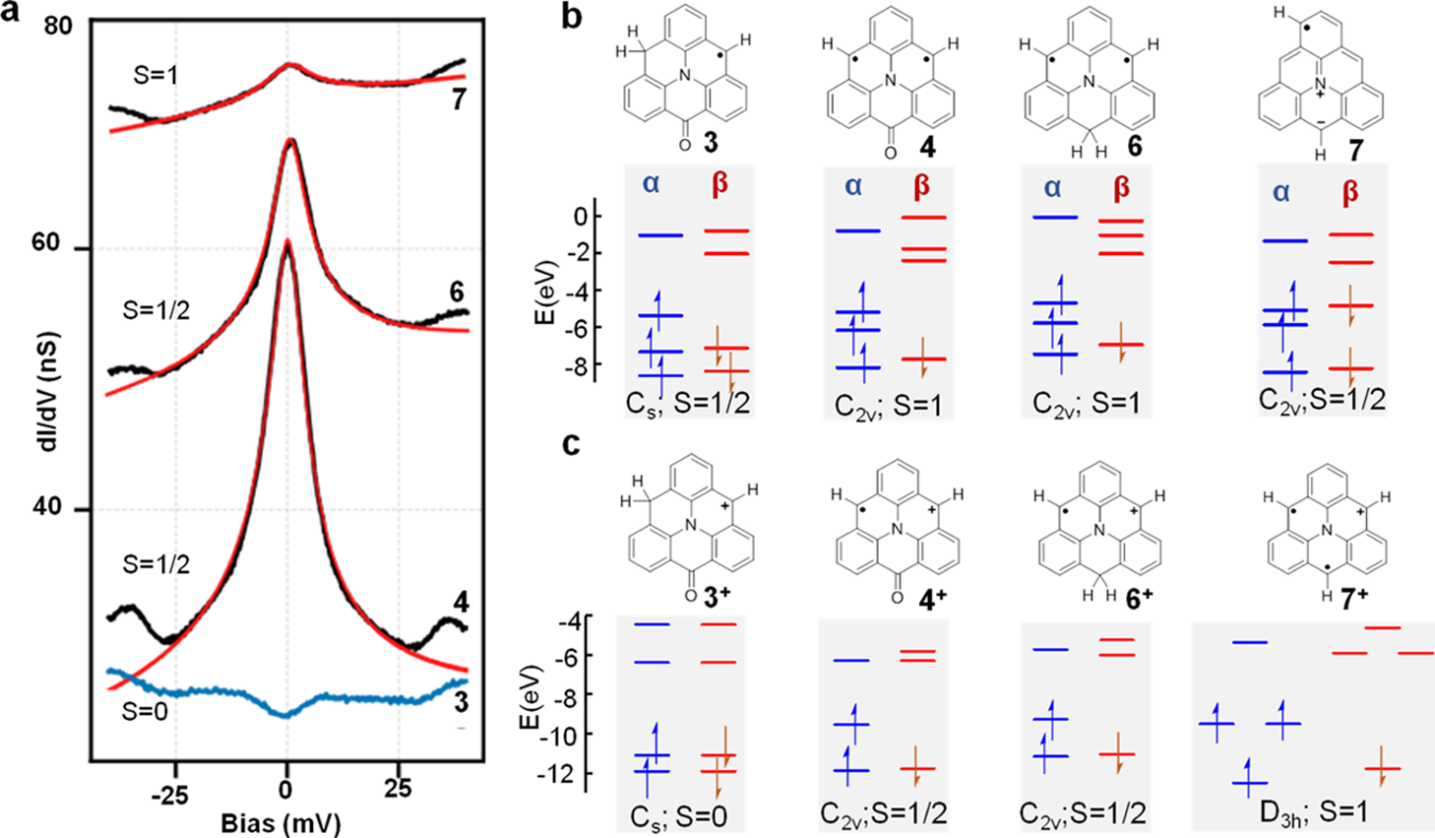
(a) Low-energy d*I*/d*V* spectra taken of molecules **3**, **4**, **6**, and **7** on Au(111). Lock-in amplitude: 2 mV. A Frota function is used to fit the spectra from **4**, **6**, and **7**, which are attributed to Kondo resonances. (b,c) DFT-calculated energy levels of frontier molecular α and β spin orbitals of the (b) neutral and (c) positively charged molecules **3**, **4**, **6**, and **7** (in vacuum). The drawn resonance structure of **7** will be further discussed in Figure 5 and its corresponding text.

Next, we investigate the magnetism of **3**, **4**, **6**, and **7**, which are all expected to be open-shell systems as predicted by DFT calculations in vacuum (Figure 3b). Figure 3a shows their corresponding low-energy d*I*/d*V* spectra on Au(111). The spectrum of **3** does not exhibit any visible signal (apart from the two well-known inelastic vibrational modes of the CO molecule at the tip apex at ∼5 and ∼35 mV), implying a closed-shell structure.^40,41^ The spectra from **4**, **6**, and **7** all exhibit symmetric zero-energy peaks around the Fermi level that are attributed to Kondo resonances,^42^ as widely reported in metal-supported open-shell carbon structures.^11^ This is further supported by the temperature-dependent spectra of intermediate **6** (Figure S8). Fitting the spectra with a Frota function^43^ reveals a rapid peak broadening with increasing temperature, following the characteristic trend of Kondo resonances,^12,13,44^ which originates from the screening of the local spin by the conduction electrons of the underlying metal substrate.^42,45^ The Kondo resonance from **4** and **6** has high and comparable amplitudes and also similar full width at half-maximum (FWHM; 8.1 ± 0.6 mV for **4** and 8.6 ± 0.1 for **6**; derived from six and two data points, respectively), while the Kondo resonance of **7** on Au(111) is much weaker and has an apparently larger FWHM (13.0 ± 2 mV; derived from six data points). This hints at **4** and **6** probably displaying a doublet ground state (*S* = 1/2), whereas **7** presumably holds a high-spin ground state (*S* ≥ 1), whose underscreened Kondo peaks typically display much lower amplitude than those from a *S* = 1/2 system.^18,37,46^

The proposed ground-state spin for the four molecules (**3**, **4**, **6**, and **7**) based on the registered Kondo resonances does not match the DFT predictions for the neutral molecules (Figure 3b). However, spin multiplicities computed for the corresponding cationic species **3**^+^ (*S* = 0, closed-shell), **4**^+^, **6**^+^ (*S* = 1/2), and **7**^+^ (*S* = 1) are in excellent agreement with the experimental characterization (Figure 3c). In other words, electron transfer from the molecule to the Au(111) substrate reconciles the experiments with the theory.

The charge transfer can be understood from the high work function of the Au(111) surface and the associated low binding energy of highest occupied molecular orbital (HOMO) levels of hydrocarbon structures atop,^47^ along with the n-doping effect of graphitic N-subsituents.^48^ Indeed, similar charge-transfer processes were observed in a number of N-doped molecules on Au(111).^49−51^ In contrast, no charge transfer was detected in the previous works studying unsubstituted extended triangulenes on Au(111).^14,15^ These findings agree with the lower ionization energy of aza-triangulene (5.0 eV, see Figure S9) as compared to those of unsubstituted triangulene and extended triangulenes (6.3 and 6.2 eV).

### Electronic Properties of Aza-Triangulene on Surfaces

**Figure 4:**
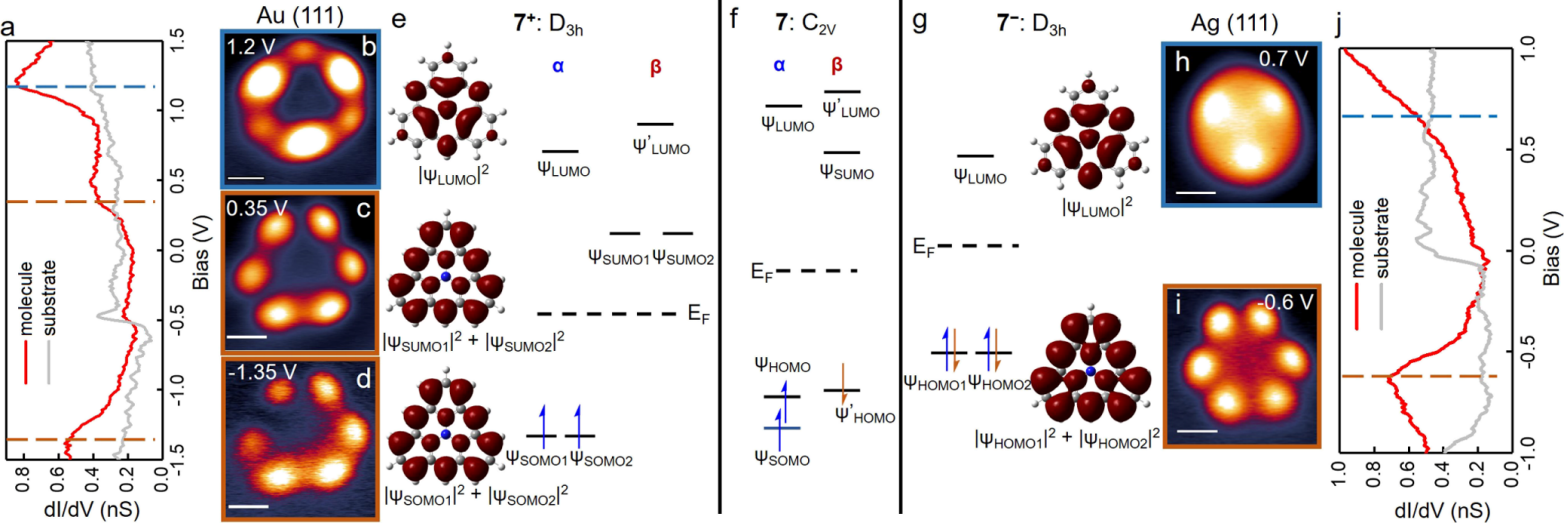
(a,g) Long-range d*I*/d*V* spectra taken on aza-triangulene on Au(111) and Ag(111), respectively. Lock-in amplitude: 20 mV. (b–d) Constant–current d*I*/d*V* maps of aza-triangulene on Au(111) using a metal tip at the energies of 1.2, 0.35, and −1.35 V, respectively. (e,g) DFT-calculated spatial distribution of electron DOSs corresponding to frontier molecular orbitals of aza-triangulene on Au(111) and Ag(111), respectively. The calculated electronic DOSs are from the summation of the squares of the calculated degenerate orbitals. (f) Energy levels of the neutral aza-triangulene. (h,i) Constant–current d*I*/d*V* maps of aza-triangulene on Au(111) using a metal tip at the energies of 0.7 and −0.6 V, respectively. All the scale bars are 5 Å.

In the following, we focus our attention to the electronic structure around the Fermi level of aza-triangulene (Figure 4). On Au(111), the d*I*/d*V* spectroscopy on **7** presents three prominent peaks at −1.35, 0.35, and 1.2 V (Figure 4a). The spatial maps of the d*I*/d*V* signal at −1.35 and 0.35 V are identical (Figure 4c,d) and can be associated to the SOMOs and SUMOs of **7**^+^, respectively, separated by a 1.7 eV Coulomb gap.^13^ Also the amplitude of the Kondo resonance appears with the same distribution as the SOMOs and SUMOs (Figure S10), supporting that the Kondo resonance originates from the SOMOs of the cationic aza-triangulene. In turn, the resonance at 1.2 V corresponds to the lowest unoccupied molecular orbital (LUMO; Figure 4b). The experimental d*I*/d*V* maps of all these orbitals exhibit threefold symmetry and match well with the corresponding DFT-calculated density of states (DOSs) for the positively charged aza-triangulene (Figure 4e; more details in Figure S11). In contrast, the conductance maps do not fit the DOS distribution of the frontier orbitals of the neutral aza-triangulene (Figure S11), corresponding to nondegenerate irreducible representations of the *C*_2*v*_ symmetry point group (Figures 4f and S11). Note that the slight asymmetry in the d*I*/d*V* maps of the frontier orbitals is probe-dependent (compare, e.g., Figure 4c,d with the conductance maps in Figure S10) but always comparable for both the occupied and unoccupied states and can thus be safely assigned to mere tip asymmetries.

It is known that the energy-level alignment in molecule–substrate dyads hinges on the substrate’s work function. Ag(111) displays a work function that is ∼0.6 eV lower than that of Au(111) (∼4.7 *vs* ∼5.3 eV),^52^ a difference that may be sufficient to prevent the electron transfer from the molecule to the substrate. Therefore, we used Ag(111) as the substrate to produce aza-triangulene by the same hydrogenation procedure as on Au(111), followed by a 300 °C annealing treatment (see Figure S12). Neither a Kondo resonance nor any spin excitation was detected for aza-triangulene on Ag(111) (Figure S12), suggesting a closed-shell ground state. This is clearly indicative that, rather than remaining as a neutral molecule with a doublet ground state, one electron was transferred from Ag(111) to aza-triangulene.^53^ According to the DFT-calculated molecular orbitals (Figure 4g), aza-triangulene indeed becomes closed-shell when it receives an extra electron and, like **7**^+^, also **7**^**–**^ recovers the threefold (*D*_3*h*_) symmetry since the doubly degenerate (e″) HOMOs are fully occupied and Jahn–Teller distortions are deactivated.

In the long-range d*I*/d*V* spectra of aza-triangulene on Ag(111) (Figure 4j), an electronic resonance at −0.6 eV is observed, along with a conductance increase starting at around 0.5 eV that we assign to HOMOs and LUMO, respectively. Additional bias-dependent conduction maps (Figure S13) reveal the best-resolved maps at −0.6 and 0.7 eV, which we thus assign to the respective orbital’s energies. Their appearance (Figure 4g,h) exhibits threefold symmetry and matches well with the DFT-calculated spatial distribution of the HOMOs and LUMO DOS of **7**^–^ (Figure 4h,i).

Although the calculated DOS of the LUMO of **7**^+^ and **7**^**–**^ looks almost identical (Figure 4e,g), the corresponding conductance maps (Figure 4b,h) differ substantially. In this respect, however, it needs to be remembered that, whereas the calculations have been performed for free-standing molecules, the experimental data are obtained on different substrates, Au(111) and Ag(111). That is, molecule–substrate hybridization effects may affect both systems differently. In addition, the orbitals have an anisotropic decay as a function of the distance to the molecular plane and the tip-sample distance during the measurements on both substrates may be notably different given the disparate energy of the LUMO.^54,55^ Both of these effects presumably account concomitantly to the observed divergences.

## Discussion

Based on above observations, charge transfer between aza-triangulene and the substrate increases the molecular symmetry (from *C*_2*v*_ to *D*_3*h*_). Whereas the neutral molecule is predicted to display a *C*_2*v*_ symmetry in its ground state, DFT calculations disclose, in agreement with experiments, that positively and negatively charged aza-triangulenes exhibit a *D*_3*h*_ symmetry instead. Moreover, while **7**^+^ holds a ferromagnetic ground state (*S* = 1), **7**^**–**^ is a closed-shell (*S* = 0) species.

**Figure 5:**
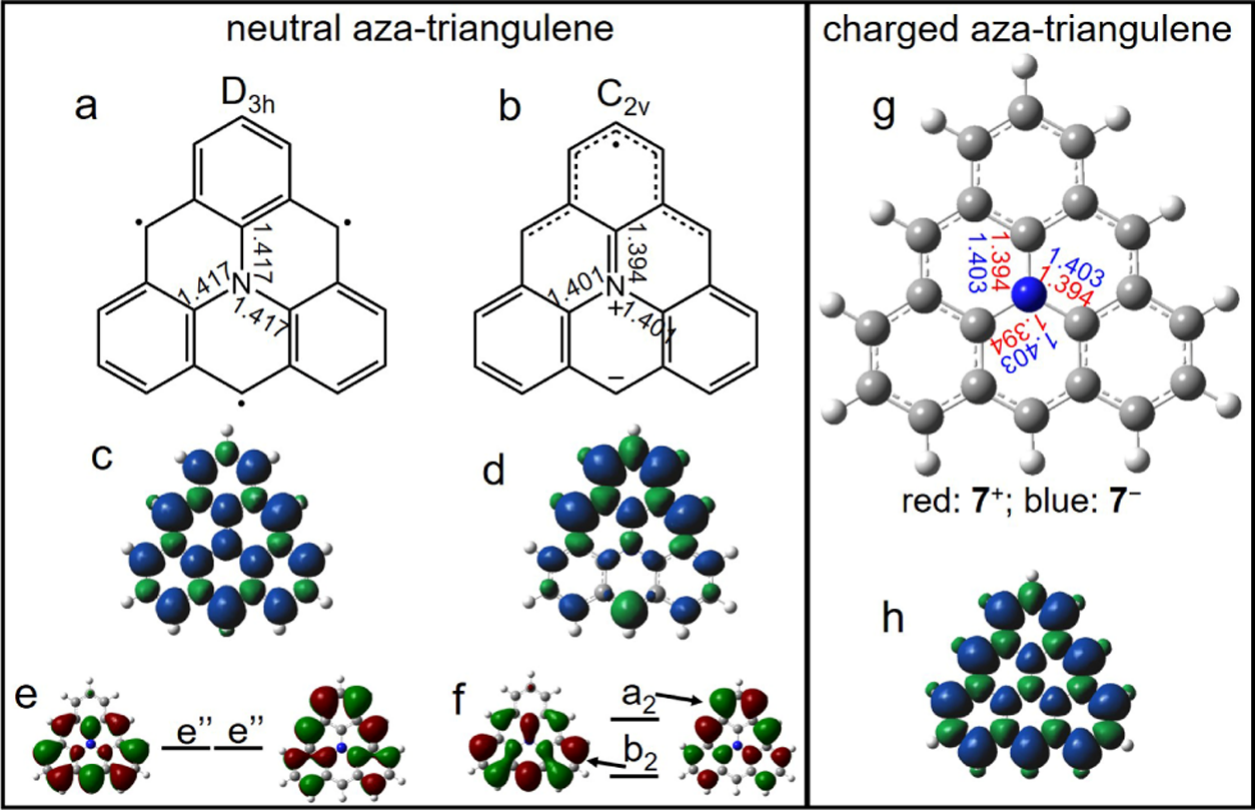
(a,b) Molecular structure of neutral aza-triangulene with *D*_3*h*_ and *C*_2*v*_ symmetry, respectively, along with the calculated length of the carbon–nitrogen bonds (Å). (c,d) Spin density distributions of neutral aza-triangulene with *D*_3*h*_ and *C*_2*v*_ symmetry, respectively. (e,f) DFT computed frontier molecular orbitals of **7** with *D*_3*h*_ and *C*_2*v*_ symmetry, respectively, along with their relative energy alignments. (g) Molecular model of charged aza-triangulenes. C–N bond lengths are indicated as red and blue numbers (Å) for positively and negatively charged cases, respectively. (h) Spin density distribution of **7**^+^ (*S* = 1).

In the following, we further rationalize the change of symmetry upon charge transfer (Figure 5). According to calculations,^27^ a neutral aza-triangulene with *D*_3*h*_ symmetry (*S* = 3/2) has longer carbon–nitrogen bond lengths (Figure 5a) than that in the *C*_2*v*_ conformation (Figure 5b). This suggests a lower bond order for the C–N bonds in the *D*_3*h*_ configuration, resulting in a resonant structure with three unpaired π radicals (Figure 5a) delocalized evenly around the threefold symmetric molecule and the nitrogen p_*z*_ orbital not participating in the conjugated molecular π-network. Note that, for this structure, the calculated spin density on N (Figure 5c) is parallel to that of the three neighboring C atoms, which goes against the antiferromagnetic alignment of electronic spins in chemical bonds and is thus in agreement with the absence of a C–N π-bond.^56^ In contrast, adoption of a *C*_2*v*_ symmetry with *S* = 1/2 (Figure 5b), driven by a Jahn–Teller distortion, reduces the C–N bond lengths (lowest with one particular neighboring carbon atom; Figure 5b). A resonance structure that may correspond to this configuration involves a zwitterionic structure and a C–N π-bond (Figure 5b). This is also supported with the calculated spin density, which reveals no spin frustration,^56^ that is, antiferromagnetic spin polarization interactions for all neighboring atoms (Figure 5d), stabilizing the *C*_2*v*_ spin doublet (*S* = 1/2) structure by 0.49 eV with respect to the *D*_3*h*_ quartet.^27^ Besides, the agreement between the calculated spin density distribution and the proposed location of the π radical also matches the suggested zwitterionic structure (Figure 5b,d). Lastly, it also reconciles the ground-state spin 1/2 with Ovchinnikov’s rule since the bonding nature of the N p_*z*_ orbital justifies its counting toward *N*_B_, whereas the carbon atom hosting the negative charge at the low side edge has its p_*z*_ orbital doubly occupied and does not count toward *N*_A_ (*N*_A_ = 11, *N*_B_ = 10, and *S* = 1/2).

However, whereas the picture above rationalizes the symmetry and net spin for the neutral species **7**, it does not explain the symmetry change upon molecular charging. This can be understood from the distinct filling of the frontier orbitals. In the *D*_3*h*_ symmetry, the twofold e″ orbitals present larger electron density probability at the molecular edges (Figure 5e), with a threefold symmetric-combined DOS. Neutral aza-triangulene has an odd number of p_z_ electrons with three electrons in the e″ orbital space, which prevents an equal orbital occupation and induces a Jahn–Teller distortion toward the *C*_2*v*_ structure (Figure 5b) lowering the overall energy of the system. Frontier orbitals of the *C*_2*v*_ structure (a_2_ and b_2_; Figure 5f) present spatial distributions similar to the e″ pair in the *D*_3*h*_ arrangement, with two electrons filling the b_2_ orbital and one in the a_2_ orbital localized on the molecular vertex crossed by the *C*_2_ axis. As a result, the twofold symmetric vertex of the molecule remains with a lower electronic density but higher spin density (Figure 5d).^57^

Positively and negatively charged aza-triangulene molecules (**7**^+^ and **7**^–^) hold an even number of p_z_ electrons, allowing for an equal population of the e″ frontier orbital pair. In **7**^+^, the e″ space is partially occupied and vibronic couplings inducing *D*_3*h*_ → *C*_2*v*_ Jahn–Teller distortion, as in neutral **7**, compete with Hund’s-rule coupling emerging from Coulomb repulsion and exchange interaction of the two electrons in the two e″ orbitals. In this case, the Jahn–Teller-induced orbital stabilization is not enough to overcome Hund’s rule and, as a result, **7**^+^ has a spin-triplet *D*_3*h*_ ground state (like its isoelectronic sister molecule pristine triangulene)^3^ with the positive charge delocalized over the whole molecule. The calculated structure reveals a reduced C–N bond length comparable to that of the neutral *C*_2*v*_ structure, hinting at the involvement of the N atom in the π-conjugation network, that is, higher bond order and π-character of the C–N bond (Figure 5g). Indeed, this is also supported by the calculated spin density (Figure 5h), which, in contrast to the neutral *D*_3*h*_ case (Figure 5c), now shows antiferromagnetic spin orientations between the N and its neighboring C atoms, as well as between all neighboring atoms throughout the whole molecule (Figure 5h).

In **7**^–^, the e″ orbitals become both doubly occupied, resulting in a closed-shell structure with threefold symmetry and no net spin. Also, here the calculated bond length for C–N (Figure 5g) is shorter than that for the neutral *D*_3*h*_ structure, suggesting again the involvement of the N atom in the π-conjugation network.

Altogether, this is a simplistic chemical view of symmetries vis-à-vis the charge state of the molecule: the neutral aza-triangulene adopts a conformation, that is, symmetry and resonance structure, that minimizes the number of unpaired spins at the expense of lowering its symmetry and generating localized charges in the zwitterionic form. In turn, the charged molecules adopt a threefold symmetric conformation that delocalizes the charge and leads to closed-shell (**7**^–^) or open-shell (**7**^+^) structures.

## Conclusions

In summary, we synthesized nitrogen-doped triangulene on both Au(111) and Ag(111) surfaces by H reduction, followed by annealing and tip manipulations. The Kondo resonances of different intermediates and products provide pieces of evidence that charge transfer sets in from molecules to the Au(111) substrate and the aza-triangulene acquires a triplet open-shell ground state. This is further confirmed by the excellent agreement between the experimentally obtained conductance maps at the energies of the frontier molecular orbitals of aza-triangulene and DFT-calculated counterparts on the positively charged aza-triangulene. Opposite from the case on Au(111), a low work-function Ag(111) substrate donates an electron to aza-triangulene, leading to the formation of a closed-shell structure. In addition, we provide a chemically intuitive picture for the origin of the *C*_2*v*_ symmetry of a neutral aza-triangulene molecule and rationalize the symmetry divergences of its charged states.

## Supporting Information Available

Methods, STM data of **1**, STM data of **1** after hydrogenation, STM data of a sample after thermally triggered dehydrogenation, STM data of ketone-functionalized molecules at various dehydrogenation stages, temperature-dependent Kondo resonance analysis of **6**, calculated ionization energies, conductance maps of **7** with Cl-functionalized probes, DFT-calculated wavefunctions of molecular orbitals of **7** in different charging states, STM data of **7** on Ag(111), and bias-dependent conductance maps of **7** on Ag(111) (PDF)
