# Supplementary material for: Aza-Triangulene: On-Surface Synthesis and Electronic and Magnetic Properties
Source: J Am Chem Soc. 2022 Mar 7;144(10):4522–9. doi: 10.1021/jacs.1c12618 (PMC8931755; doi:10.1021/jacs.1c12618)
Supplement: Supplementary file 1 — ja1c12618_si_001.pdf [file ja1c12618_si_001.pdf]

# Supporting information

# Aza-Triangulene: On-Surface Synthesis, Electronic and Magnetic Properties

Tao Wang,[1,2,‡,*] Alejandro Berdonces-Layunta,[1,2,‡] Niklas Friedrich,[3] Manuel Vilas-Varela,[4] Jan Patrick Calupitan,[2,*] Jose Ignacio Pascual,[3,5] Diego Peña,[4] David Casanova,[1,5] Martina Corso,[1,2] and Dimas G. de Oteyza[1,2,5,6,*]

[1]Donostia International Physics Center, 20018 San Sebastián, Spain

[2]Centro de Fisica de Materiales CFM/MPC, CSIC-UPV/EHU, 20018 San Sebastián, Spain

[3]CIC nanoGUNE BRTA, 20018 San Sebastián, Spain

[4]Centro Singular de Investigación en Química Biolóxica e Materiais Moleculares (CiQUS) and Departamento de Química Orgánica, Universidade de Santiago de Compostela, 15782 Santiago de Compostela, Spain

[5]Ikerbasque, Basque Foundation for Science, 48009 Bilbao, Spain

[6]Nanomaterials and Nanotechnology Research Center (CINN), CSIC-UNIOVI-PA; 33940 El Entrego, Spain

* taowang@dipc.org; janpatrick.calupitan@ehu.eus; d.g.oteyza@cinn.es

# Experimental

## STM Experiments

STM measurements were performed using a commercial Scienta-Omicron LT-STM at 4.3 K. The system consists of a preparation chamber with a typical pressure in the low $10^{-10}$ mbar regime and a STM chamber with a pressure in the $10^{-11}$ mbar range. The Au(111) and Ag(111) crystals were cleaned *via* two cycles of $Ar^+$ sputtering and annealing (720 K for Au and 700 K for Ag). Ketone substituted triangulene precursor molecule was evaporated from a home-built evaporator at 460 K. Hydrogenation of the sample was achieved with a hydrogen cracking source with a leak valve. The preparation chamber was first filled to a pressure of $2 \times 10^{-7}$ mbar, after which the tungsten tube was heated to around 2800 K with a heating power of 80 W. The sample was then placed in front of the source for 2 minutes.

All STM and STS measurements were performed at 4.3 K. To obtain BR-STM images, the tip was functionalized with a CO molecule that was picked up from the metal surfaces. CO was deposited onto the sample *via* a leak valve at a pressure of approximately $5\times10^{-9}$ mbar and a maximum sample temperature of 7.0 K. CO can be picked up with a metallic tip by scanning with a high current and negative bias (*e.g.* I=1 nA, U=−0.5 V). Functionalization of the tip with a Cl atom is achieved by approaching the tip by 350 pm from initial stabilization conditions of 100 pA and 100 mV with the feedback off, on the top of the deposited NaCl island on Au(111).[1] After pickup, an increase in resolution is seen and a vacancy in the NaCl island can also be observed, which was reported in detail in our previous work.[2] dI/dV measurements were recorded with the internal lock-in of the system. The oscillation frequency used in experiments is 797 Hz. The amplitude for each spectrum is shown in Figure captions.

## DFT calculations

DFT calculations were performed by the Gaussian 16 package[3] using the M06-2X functional and 6-311G(d,p) basis set. Results were visualized by using the software Gaussview[4] and the squares of the wavefunction were generated using the cubman module.

For the positively-charged species, the atomic positions of the neutral N-doped triangulene[5] were used as input for the optimization. Initial optimization set to charge=+1 and spin=1 yielded a $D_{3h}$ geometry, independent of whether the starting geometries corresponded to $D_{3h}$ or $C_{2v}$ geometry. Similarly, starting from any of the neutral $C_{2v}$ or $D_{3h}$ geometries led to a $D_{3h}$ geometry for the negatively-charged species. All geometries and symmetries were confirmed further by re-performing calculations with tight convergence criteria using the keywords *opt=tight int=ultrafine*. Ionization energies (Figure S9) were obtained as the electronic energy difference between potential energy surface minima of neutral and cationic species.

## Solution synthesis

Starting materials were purchased from TCI and Sigma-Aldrich and used without further purification. O,O',O''-Amino-trisbenzoic acid-trimethylester (**S3**) and 4H-benzo[9,1]quinolizino[3,4,5,6,7-defg]acridine-4,8,12-trione (**1**) were synthesized following slightly modified literature procedures.[6,7] Reactions were carried out in flame-dried glassware and under an inert atmosphere (Ar) using Schlenk techniques. Thin-layer chromatography (TLC) was performed on Silica Gel 60 F-254 plates (Merck). Column chromatography was performed on silica gel (40-60 µm). NMR spectra were recorded on a Varian Mercury 300 spectrometer.

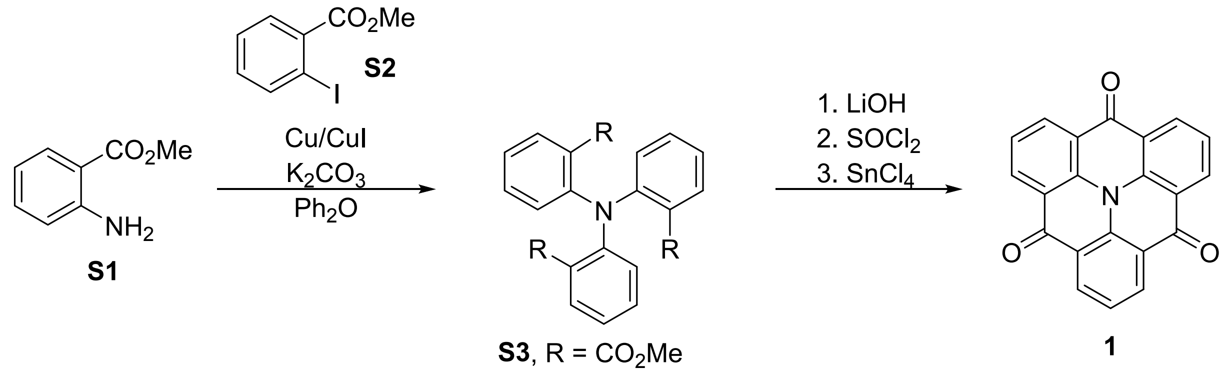


**Figure S1.** Synthesis of compound **1**.

**Synthesis of compound S3.** A mixture of methyl anthranilate (**S1**) (2.0 ml, 15.5 mmol), methyl 2-iodobenoate (**S2**) (6.5 ml, 43.3 mmol), $K_2CO_3$ (5.3 g, 38.8 mmol), Cu (0.2 g, 3.1 mmol) and CuI (0.3 g, 1.6 mmol) in diphenylether (15 ml) was heated at 190 °C under Ar for 72 hours. The solvent was removed under reduced pressure and the residue was purified by column chromatography ($SiO_2$; 4:1 hexane/ethyl acetate) to afford **S3** (3.6 g, 55%) as a yellow solid. $^1H$ NMR ($CDCl_3$) δ 7.60 (m, 3H), 7.36 (m, 3H), 7.07 (m, 6H), 3.37 (s, 9H) ppm.

**Synthesis of compound 1.** A mixture of **S3** (0.50 g, 1.2 mmol) and $LiOH.H_2O$ (0.90 g, 21 mmol) in THF: $H_2O$ (4:1, 25 mL) was refluxed for 6 h. After cooling to room temperature, the mixture was diluted with ethyl acetate (10 mL) and water (10 mL), and the phases were separated. The aqueous phase was acidified with concentrated HCl until pH = 2 and then extracted with ethyl acetate (3x10 mL). Organic extracts were combined, dried over anhydrous $Na_2SO_4$, filtered and evaporated. The crude product was dissolved in dichloromethane (20 mL) and two drops of DMF were added, followed by $SOCl_2$ (1.7 mL). The resulting mixture was refluxed for 3 h and then cooled to 0 ºC. Then, $SnCl_4$ (1.7 mL) was added dropwise. The resulting mixture was refluxed for 16 h and the formed precipitate was collected by filtration and washed with methanol (2x20 mL). The obtained solid was then suspended in 1 M NaOH (20 mL) and stirred for 30 min, filtered, and washed with water (3x20 mL), methanol (2x20 mL) and acetone (2x20 mL) to afford **1** (115 mg, 45%) as a grey solid. $^1H$-NMR (10% TFA in $CDCl_3$) δ 9.19 (d, J = 7.7 Hz, 6H), 8.05 (t, J = 7.8 Hz, 3H). ppm.

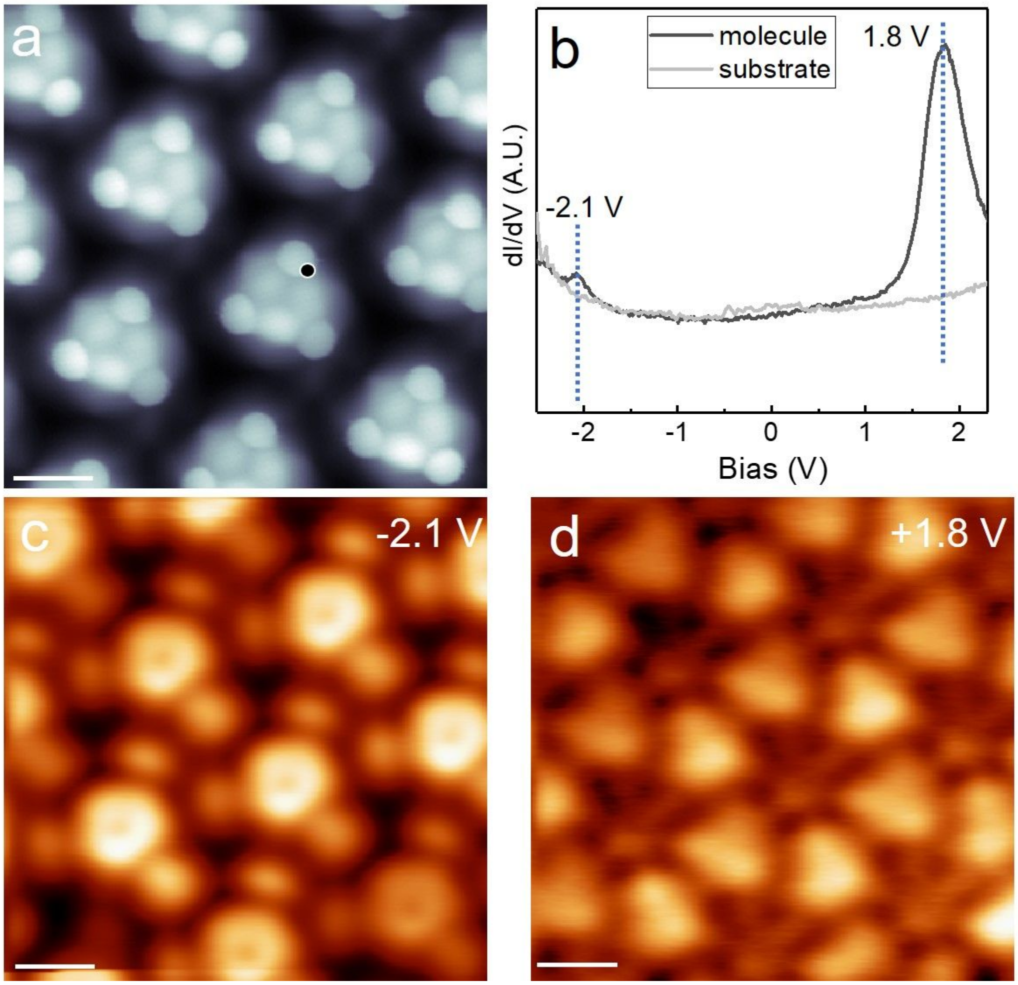


**Figure S2**. (a) BR-STM image of ketone substituted N-triangulenes on Au(111). (b) dI/dV spectra taken on N-triangulene and the Au(111) substrate with a CO-functionalized probe, respectively. The dI/dV spectroscopy on ketone substituted N-triangulene (the position is marked in (a)) presents two prominent peaks, at −2.1 and 1.8 V. (c,d) dI/dV maps taken at −2.1 and 1.8 V. All the scale bars are 5 Å.

dI/dV maps at −2.1 and 1.8 V should be associated with the HOMO and LUMO of ketone substituted N-triangulene respectively, in agreement with previous work.[8]

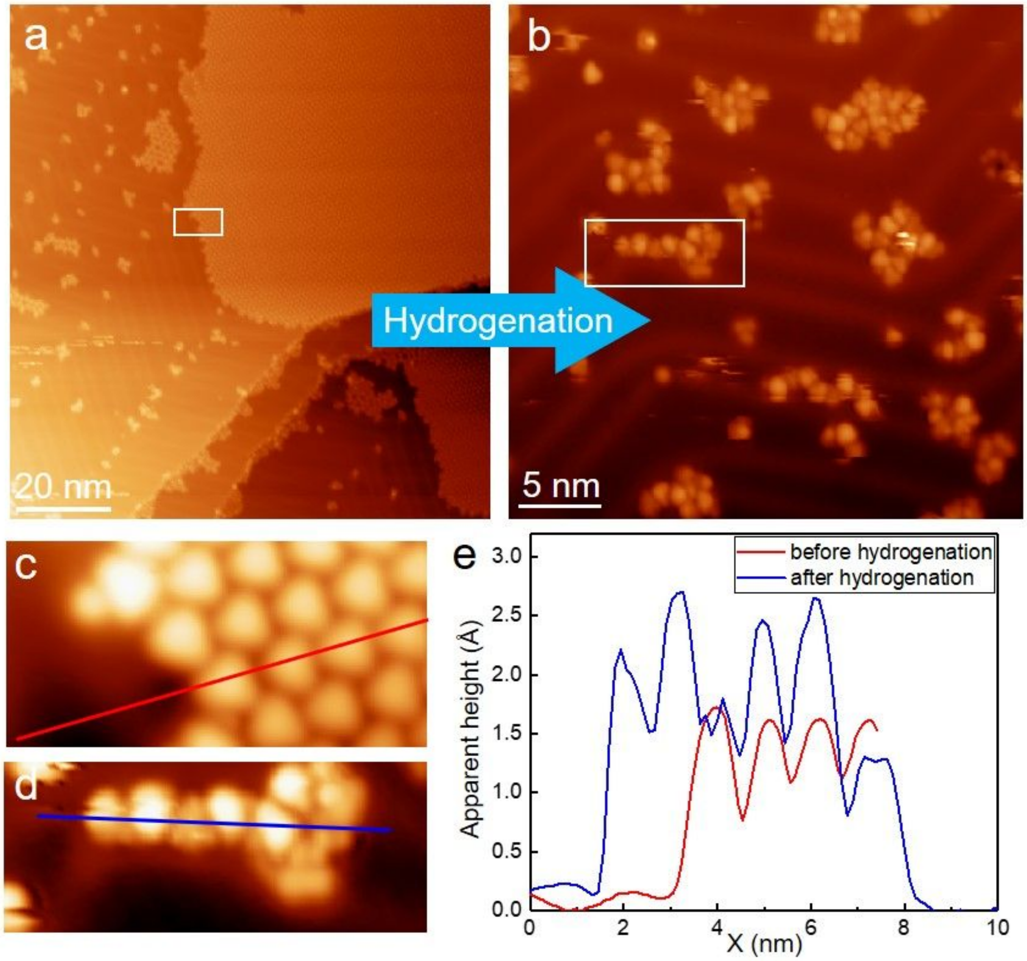


**Figure S3.** (a) Large-area STM image of the ketone substituted N-triangulene covered Au(111) sample. (b) STM image recorded after H reduction of (a). (c,d) Zoom-in STM image of the white framed region in (a) and (b) respectively. (e) Relative apparent height profiles of the lines shown in (c) and (d). The hydrogenated molecules are much higher than molecules before hydrogenation (~2.7 Å *vs.*~1.6 Å), indicating the existence of $sp^3$ carbons after hydrogenation. Scanning parameters: (a) U=1 V, I= 100 pA; (b-d) U=−1 V, I=−100 pA; metal probes.

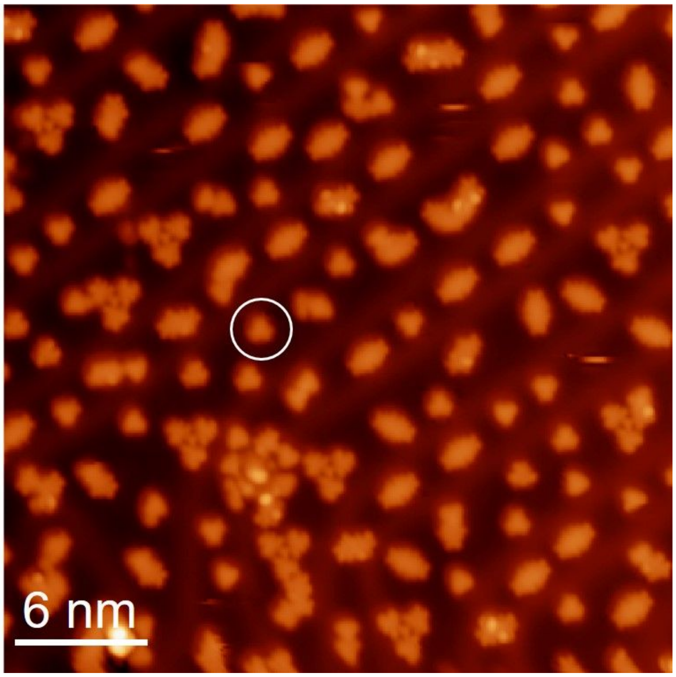


**Figure S4**. STM image of the Au(111) sample prepared by annealing a hydrogenated sample to 300 ºC. Almost all the molecules (>95%) are planarized at these conditions. The target product N-triangulene is directly obtained, as exampled by the white circle marked one. Scanning parameters: (a) U=−1 V, I=−100 pA, metal tip.

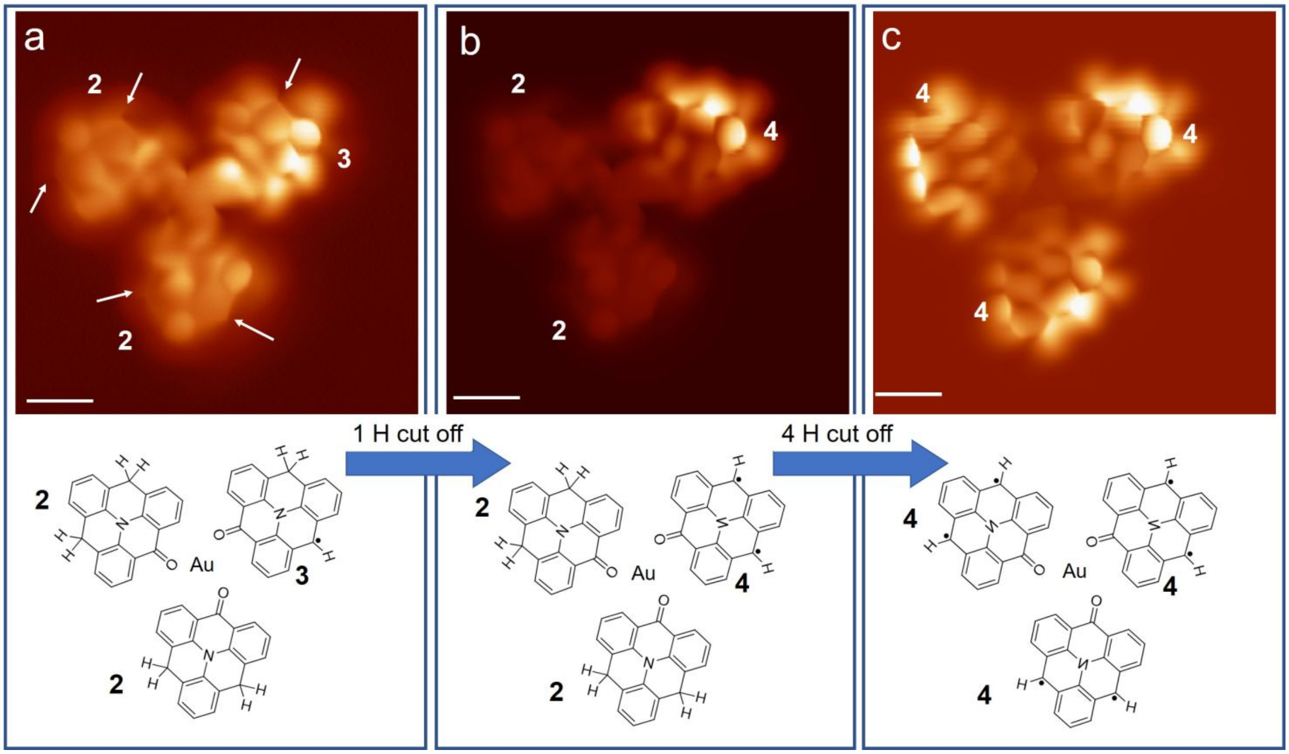


**Figure S5**. Tip manipulations on a Au(111)-supported trimer composed of two **2** and one **3**. (a) BR-STM image of the trimer structure. One monomer has one additional hydrogen while other two have two additional hydrogens, as illustrated in the chemical structures below. The rings with $sp^3$ carbons are obviously much larger than other rings, as pointed by white arrows. From (a) to (b), we cut off the additional hydrogens on **3**, producing **4**. From (b) to (c), we cut off all four remaining hydrogens and all the three monomers correspond to molecule **4**. Note that middle oxygen cannot be removed by the conventional tip manipulations. Scanning parameters: (a-c) U=5 mV, CO-functionalized probes. All the scale bars are 5 Å.

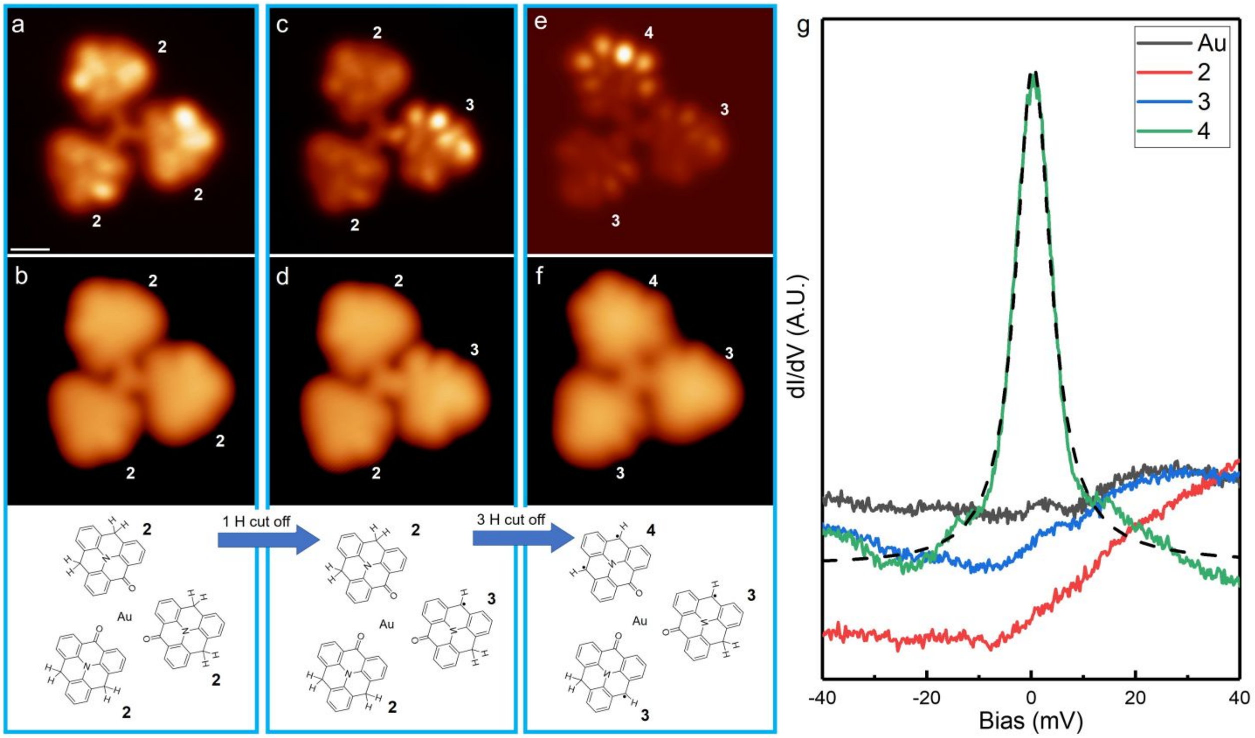


**Figure S6**. Tip manipulations on another Au(111)-supported trimer composed of three **2** to generate **3** and **4**. A Cl tip was used in all these processes. Molecular chemical structures are illustrated below the corresponding STM images. (a,b) Constant-height and constant-current STM images, showing the trimer composed of three **2**. The scale bar in (a) is 5 Å. (b,c) Constant-height and constant-current STM images taken after one H was removed (producing **3**). An obvious difference between (a) and (c), or (b) and (d) can be recognized, though without BR-STM image. (e,f) Constant-height and constant-current STM images taken after further tip manipulations which obtained two **3** and one **4**. The difference between **3** and **4** is also clearly visible. (f) dI/dV spectra taken on Au substrate, **2**, **3**, and **4**, respectively. The Kondo resonance is only observed on **4**. It is fitted by a Frota function (black dotted line) and a FWHM of 8.12±0.10 mV is obtained, in excellent agreement with the value in Figure 2 (8.1±0.6 mV). Scanning parameters: (a,c,e) U=5 mV; (b,d) U=40 mV, I=100 pA; (f) U=1 V, I =100 pA.

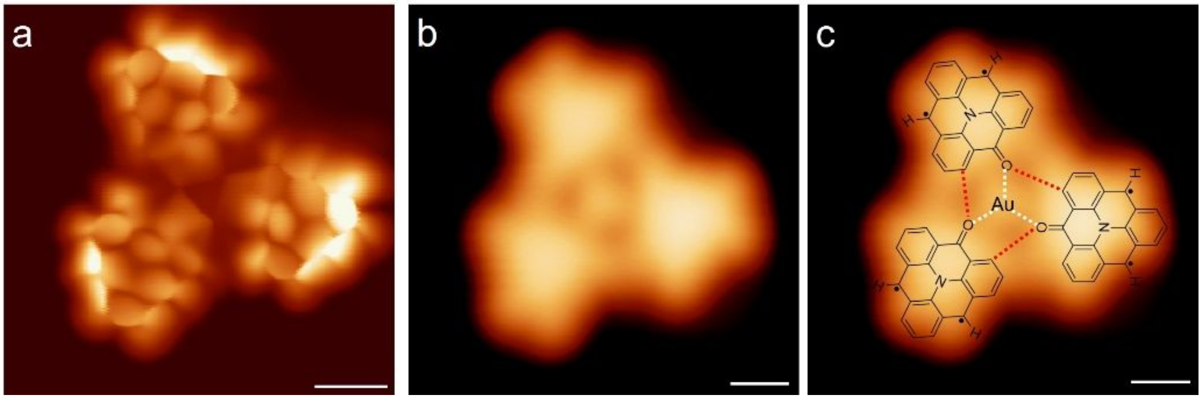


**Figure S7**. High-resolution STM images showing the Au adatom inside the trimer structure. (a) BR-STM image of a trimer structure composed of **4**. (b) shows the constant-current STM image of (a), where the middle Au adatom is clearly visible. The corresponding chemical structure matches the STM image in (b), as revealed in (c). White and red dotted lines present O···Au coordination interactions and hydrogen bonds respectively. Scanning parameters: (a) U=5 mV; (b,c) U=−0.5 V, I=−100 pA; CO-functionalized probe. All the scale bars are 5 Å.

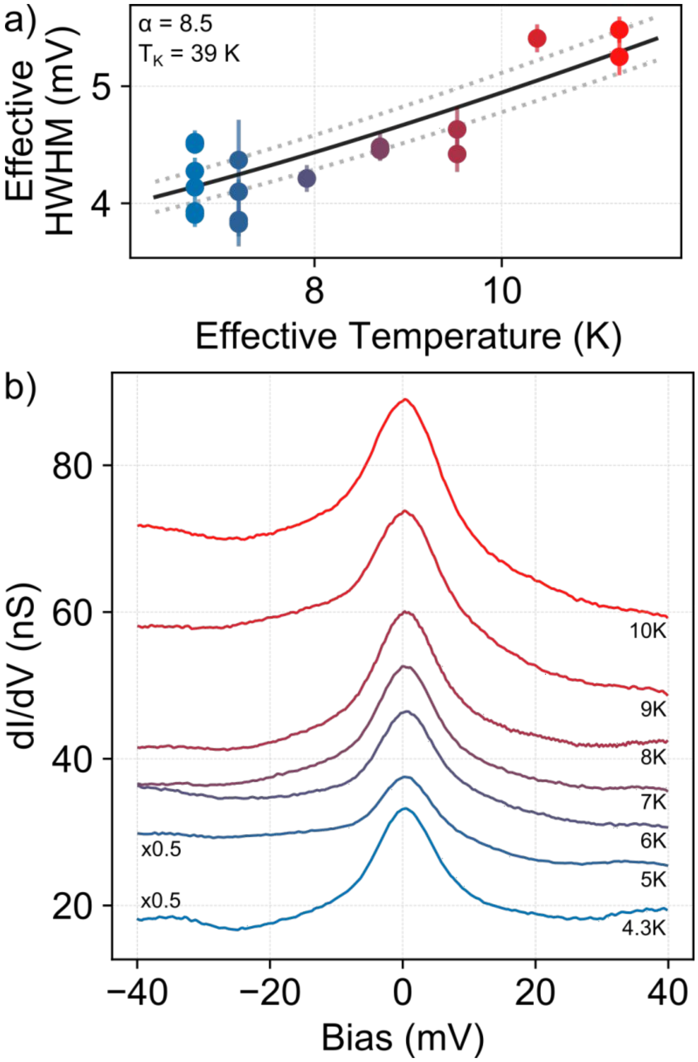


**Figure S8**. (a) Fermi liquid model of the Kondo resonance of molecule **6** on Au(111). (b) Example raw data at the different temperatures used for (a). Following Mishra *et al.*,[9] we extract the linewidth of the Kondo resonance by fitting a Frota function to the experimental data and correct the extracted half width at half maximum (HWHM) to an effective $HWHM_{eff}$ to account for finite temperature of the tip[10] at each temperature point. An effective temperature $T_{eff}$ is used to account for the modulation of the lock-in amplifier.[11] Finally, we extract the Kondo temperature *via* the empirical found formula $HWHM_{eff} = \frac{1}{2}\sqrt{(\alpha k_B T_{eff})^2 + (2 k_B T_K)^2}$ ,[12] with the Boltzmann constant $k_B$ and the fitting parameters α and $T_K$. The values α= 8.5±0.3 and $T_K$ = 39±1 K are obtained.

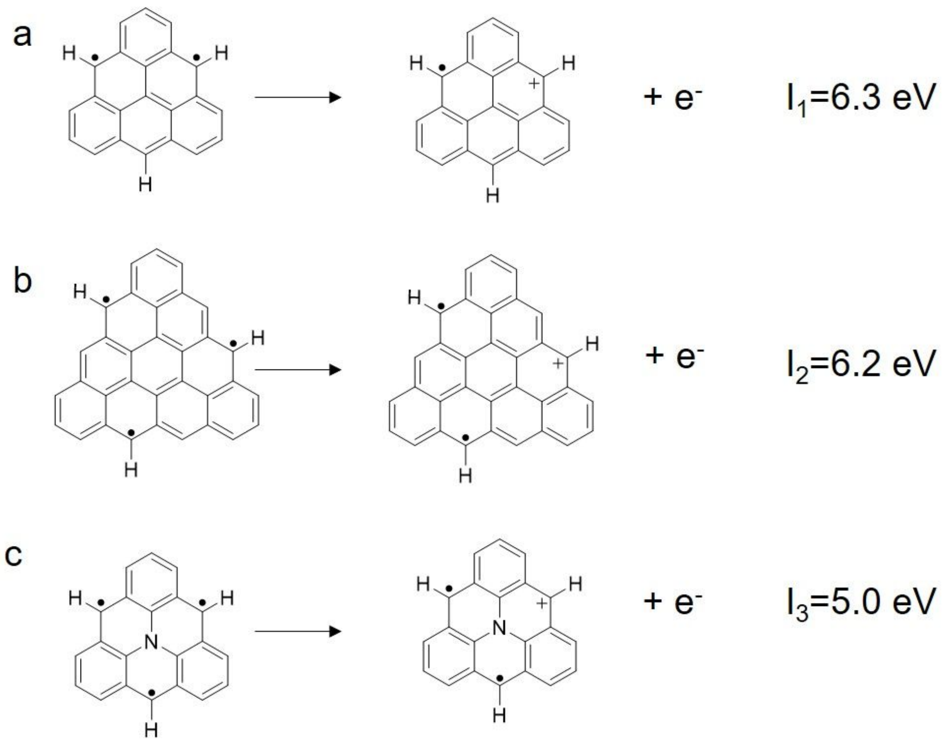


**Figure S9**. The ionization energies of (a) triangulene, (b) [4]triangulene, and (c) N-triangulene computed at the M06-2X/6-311G(d,p) level.

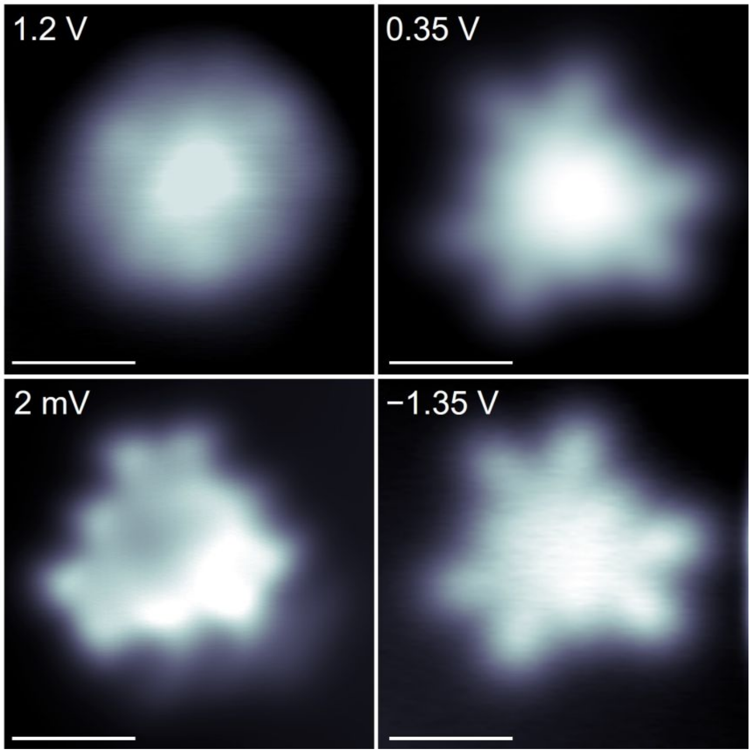


**Figure S10**. Constant-height dI/dV maps corresponding to LUMO, SUMOs, SOMOs and Kondo resonance (at 2 mV) of N-triangulene on Au(111) using a Cl-functionalized probe. All the scale bars are 5 Å.

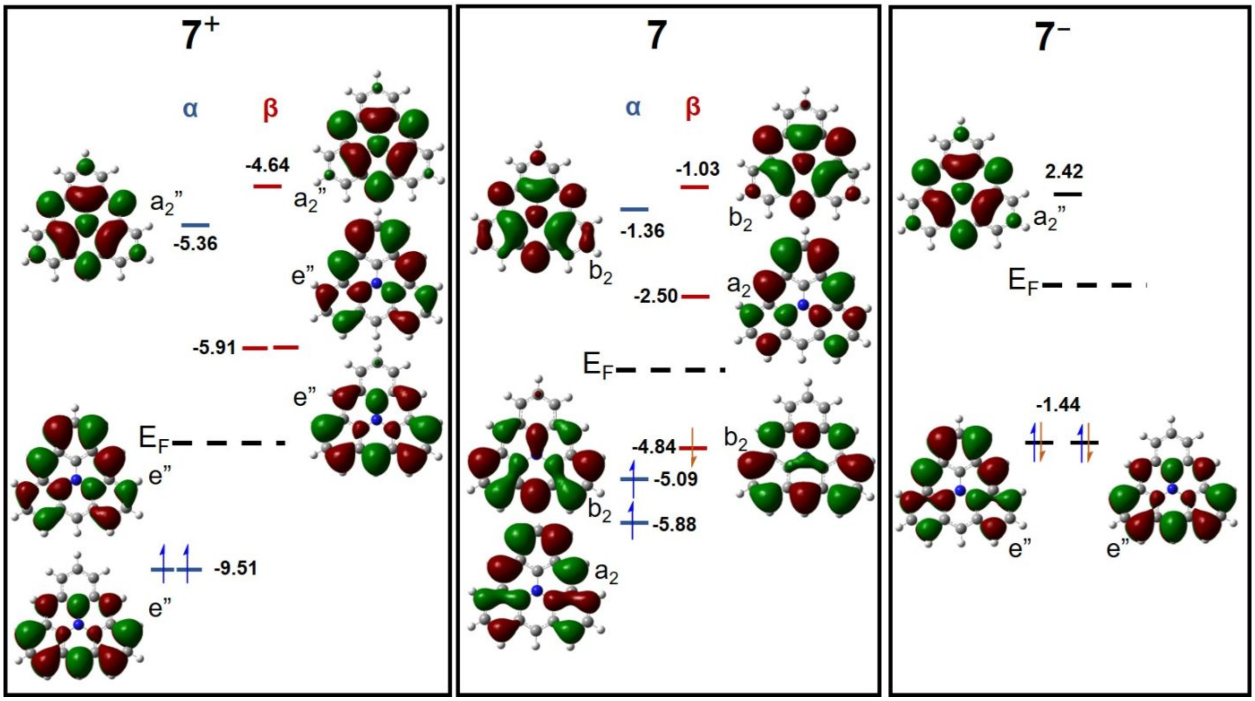


**Figure S11**. DFT calculated wave functions and energy levels of the positively charged, neutral, and negatively charged N-triangulenes, along with their corresponding energies and orbital symmetry.

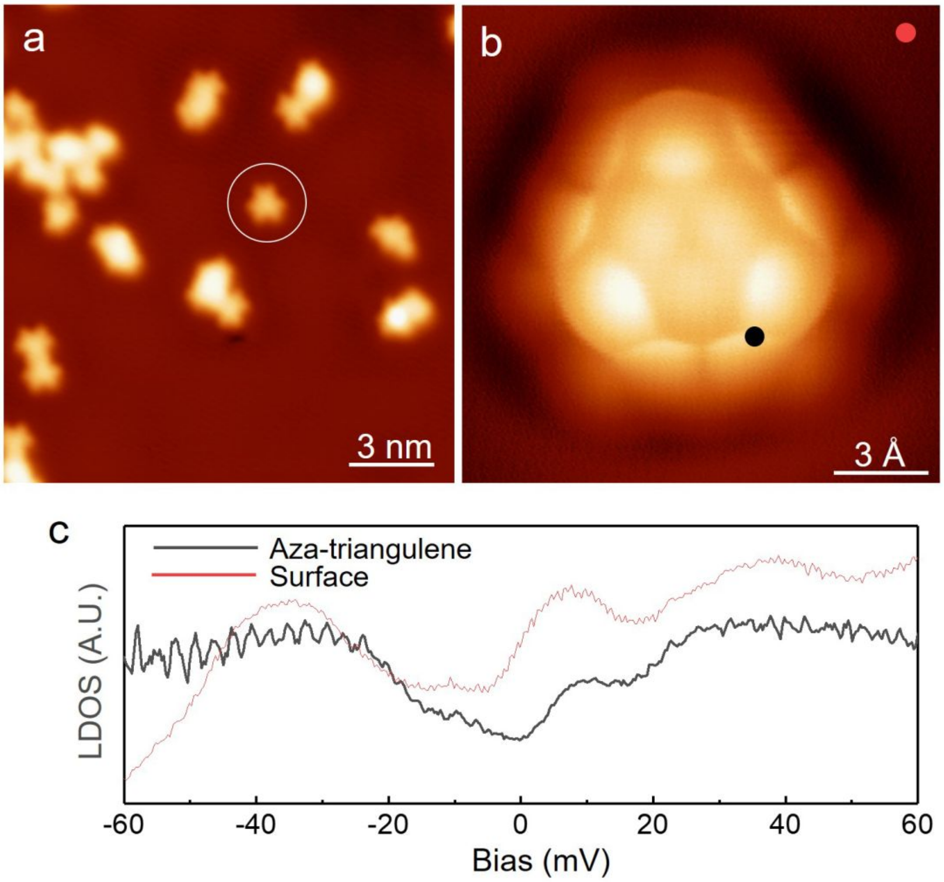


**Figure S12**. (a) STM image showing different molecular products obtained by annealing the hydrogenated ketone-N-triangulene at 300 °C on the Ag(111) sample. N-triangulene as marked by white circle coexist with some polymers generated from the fusion of N-triangulenes. Scanning parameters: U=−0.8 V; I=−80 pA. (b) Constant-height BR-STM image of N-triangulene on Ag(111) at 5 mV, using a CO-functionalized tip. (c) Low-energy dI/dV spectra taken on N-triangulene and the bare Ag(111) substrate (the positions are marked in (b)), respectively.

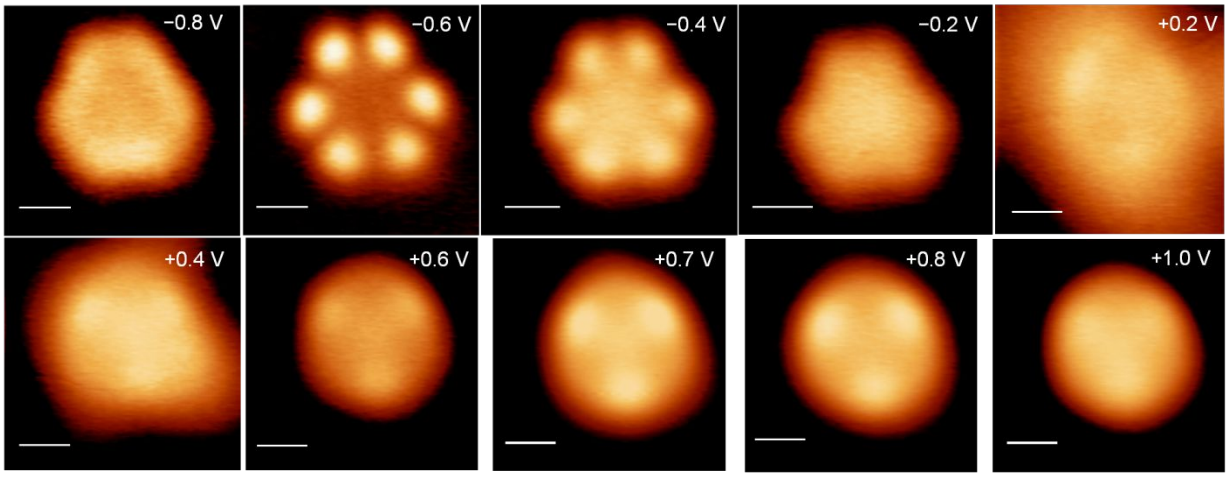


**Figure S13**. Constant current dI/dV maps at different energies using a metal tip on Ag(111). Tunneling current for the images starting from lower to higher bias values correspond to: -700pA, -500pA, -500pA, -300pA, 300pA, 500pA, 500pA, 1nA, 700pA, 900pA. All scale bars are 5 Å.

## References


(1) Mohn, F.; Schuler, B.; Gross, L.; Meyer, G. Different Tips for High-Resolution Atomic Force Microscopy and Scanning Tunneling Microscopy of Single Molecules. *Appl. Phys. Lett.* **2013**, *102* (7), 073109. https://doi.org/10.1063/1.4793200.

(2) Lawrence, J.; Brandimarte, P.; Berdonces-Layunta, A.; Mohammed, M. S. G.; Grewal, A.; Leon, C. C.; Sánchez-Portal, D.; de Oteyza, D. G. Probing the Magnetism of Topological End States in 5-Armchair Graphene Nanoribbons. *ACS Nano* **2020**, *14* (4), 4499–4508. https://doi.org/10.1021/acsnano.9b10191.

(3) *Gaussian 16, Revision C.01, Frisch, M. J.; Trucks, G. W.; Schlegel, H. B.; Scuseria, G. E.; Robb, M. A.; Cheeseman, J. R.; Scalmani, G.; Barone, V.; Petersson, G. A.; Nakatsuji, H.; Li, X.; Caricato, M.; Marenich, A. V.; Bloino, J.; Janesko, B. G.; Gomperts, R.; Mennucci, B.; Hratchian, H. P.; Ortiz, J. V.; Izmaylov, A. F.; Sonnenberg, J. L.; Williams-Young, D.; Ding, F.; Lipparini, F.; Egidi, F.; Goings, J.; Peng, B.; Petrone, A.; Henderson, T.; Ranasinghe, D.; Zakrzewski, V. G.; Gao, J.; Rega, N.; Zheng, G.; Liang, W.; Hada, M.; Ehara, M.; Toyota, K.; Fukuda, R.; Hasegawa, J.; Ishida, M.; Nakajima, T.; Honda, Y.; Kitao, O.; Nakai, H.; Vreven, T.; Throssell, K.; Montgomery, J. A., Jr.; Peralta, J. E.; Ogliaro, F.; Bearpark, M. J.; Heyd, J. J.; Brothers, E. N.; Kudin, K. N.; Staroverov, V. N.; Keith, T. A.; Kobayashi, R.; Normand, J.; Raghavachari, K.; Rendell, A. P.; Burant, J. C.; Iyengar, S. S.; Tomasi, J.; Cossi, M.; Millam, J. M.; Klene, M.; Adamo, C.; Cammi, R.; Ochterski, J. W.; Martin, R. L.; Morokuma, K.; Farkas, O.; Foresman, J. B.; Fox, D. J. Gaussian, Inc., Wallingford CT, 2016.*

(4) *GaussView, Version 6, Dennington, Roy; Keith, Todd A.; Millam, John M. Semichem Inc., Shawnee Mission, KS, 2016.*

(5) Sandoval-Salinas, M. E.; Carreras, A.; Casanova, D. Triangular Graphene Nanofragments: Open-Shell Character and Doping. *Phys. Chem. Chem. Phys.* **2019**, *21* (18), 9069–9076. https://doi.org/10.1039/C9CP00641A.

(6) Fang, Z.; Teo, T.-L.; Cai, L.; Lai, Y.-H.; Samoc, A.; Samoc, M. Bridged Triphenylamine-Based Dendrimers: Tuning Enhanced Two-Photon Absorption Performance with Locked Molecular Planarity. *Org. Lett.* **2009**, *11* (1), 1–4. https://doi.org/10.1021/ol801238n.

(7) Field, J. E.; Venkataraman, D. HeterotriangulenesStructure and Properties. *Chem. Mater.* **2002**, *14* (3), 962–964. https://doi.org/10.1021/cm010929y.

(8) Steiner, C.; Gebhardt, J.; Ammon, M.; Yang, Z.; Heidenreich, A.; Hammer, N.; Görling, A.; Kivala, M.; Maier, S. Hierarchical On-Surface Synthesis and Electronic Structure of Carbonyl-Functionalized One- and Two-Dimensional Covalent Nanoarchitectures. *Nat Commun* **2017**, *8* (1), 14765. https://doi.org/10.1038/ncomms14765.

(9) Mishra, S.; Beyer, D.; Eimre, K.; Kezilebieke, S.; Berger, R.; Gröning, O.; Pignedoli, C. A.; Müllen, K.; Liljeroth, P.; Ruffieux, P.; Feng, X.; Fasel, R. Topological Frustration Induces Unconventional Magnetism in a Nanographene. *Nat. Nanotechnol.* **2020**, *15* (1), 22–28. https://doi.org/10.1038/s41565-019-0577-9.

(10) Zhang, Y.; Kahle, S.; Herden, T.; Stroh, C.; Mayor, M.; Schlickum, U.; Ternes, M.; Wahl, P.; Kern, K. Temperature and Magnetic Field Dependence of a Kondo System in the Weak Coupling Regime. *Nat Commun* **2013**, *4* (1), 2110. https://doi.org/10.1038/ncomms3110.

(11) Girovsky, J.; Nowakowski, J.; Ali, Md. E.; Baljozovic, M.; Rossmann, H. R.; Nijs, T.; Aeby, E. A.; Nowakowska, S.; Siewert, D.; Srivastava, G.; Wäckerlin, C.; Dreiser, J.; Decurtins, S.; Liu, S.-X.; Oppeneer, P. M.; Jung, T. A.; Ballav, N. Long-Range Ferrimagnetic Order in a Two-Dimensional Supramolecular Kondo Lattice. *Nature Communications* **2017**, *8* (1), 15388. https://doi.org/10.1038/ncomms15388.

(12) Nagaoka, K.; Jamneala, T.; Grobis, M.; Crommie, M. F. Temperature Dependence of a Single Kondo Impurity. *Phys. Rev. Lett.* **2002**, *88* (7), 077205. https://doi.org/10.1103/PhysRevLett.88.077205.
